# Gonorrhœa and Homœopathy

**Published:** 1891-01

**Authors:** Robert Farley

**Affiliations:** Phœnixville, Pa.


### GONORRHŒA AND HOMŒOPATHY.

Editors Homœopathic Physician:

I want to present the "other side," as I see it, by asking Dr. T. F. Allen a few questions, suggested by his statement in the December number of your journal.

(1.) Is it certain the sufferer from gonorrhœa for eighteen months received the homœopathic remedy? that is, medicine from a homœopathic physician is not always the *similar* remedy, is it? Is it not possible that the physician failed, be he ever so skilful?

(2.) Do not all acute diseases attack persons "in apparently perfect health"? and may not the cause of scarlatina be "considered a poison" just as reasonably as the gonorrhœal virus? Why not treat the conditions of throat and skin in scarlatina as only "local manifestations" of the poison, for they are exposed to the poison when receiving the infection just as surely as the urethra is when receiving the gonorrhœal infection, are they not? Do we not meet cases of only a "few days" duration and others of several months' duration in other troubles than gonorrhœa? Does not the homœopathic remedy cause the vital energy to *react* toward health? and we are not doing the "best for them" (the patients) if we bring about this reaction from the sick state? Can "other doctors" do better than this? If your object is to "cleanse" the urethra, why use a solution of a drug? Let error be corrected!

ROBERT FARLEY.

PHŒNIXVILLE, PA., Dec. 9th, 1890.

---

